# Vaccination Rates, Perceptions, and Information Sources Used by People With Inflammatory Arthritis

**DOI:** 10.1002/acr2.11525

**Published:** 2023-01-18

**Authors:** Andrea Lyon, Alannah Quinlivan, Susan Lester, Claire Barrett, Samuel L. Whittle, Debra Rowett, Rachel Black, Premarani Sinnathurai, Lyn March, Rachelle Buchbinder, Catherine L. Hill

**Affiliations:** ^1^ The Queen Elizabeth Hospital, Woodville South, and The University of Adelaide Adelaide South Australia Australia; ^2^ The Queen Elizabeth Hospital Woodville South South Australia Australia; ^3^ Redcliffe Hospital, Redcliffe, and The University of Queensland Queensland Australia; ^4^ University of South Australia (UniSA) and Drug and Therapeutics Information Service, Southern Adelaide Local Health Network (SALHN) South Australia Australia; ^5^ The Queen Elizabeth Hospital, Woodville South, and The University of Adelaide, Adelaide, and Royal Adelaide Hospital Adelaide South Australia Australia; ^6^ The University of Sydney, Camperdown, and Royal North Shore Hospital, St Leonards, and NPS MedicineWise New South Wales Australia; ^7^ The University of Sydney, Camperdown, and Royal North Shore Hospital St Leonards New South Wales Australia; ^8^ Monash University and Monash Department of Clinical Epidemiology and Musculoskeletal Health and Cabrini Health Melbourne Victoria Australia

## Abstract

**Objective:**

To determine vaccination rates, perceptions, and information sources in people with inflammatory arthritis.

**Methods:**

Participants enrolled in the Australian Rheumatology Association Database were invited to participate in an online questionnaire, conducted in January 2020, prior to the COVID‐19 pandemic. Included questions were about vaccination history, modified World Health Organization Vaccination Hesitancy Scale, views of the information sources consulted, the Beliefs About Medicines Questionnaire, education, and the Single‐Item Health Literacy Screener.

**Results:**

Response rate was 994 of 1498 (66%). The median age of participants was 62 years, with 67% female. Self‐reported adherence was 83% for the influenza vaccine. Participants generally expressed positive vaccination views, particularly regarding safety, efficacy, and access. However, only 43% knew which vaccines were recommended for them. Vaccine hesitancy was primarily attributable to uncertainty and a perceived lack of information about which vaccines were recommended. Participants consulted multiple vaccination information sources (median 3, interquartile range 2‐7). General practitioners (89%) and rheumatologists (76%) were the most frequently used information sources and were most likely to yield positive views. Negative views of vaccination were most often from internet chatrooms, social media, and mainstream media. Factors of younger age, male gender, and having more concerns about the harms and overuse of medicines in general were associated with lower adherence and greater uncertainty about vaccinations, whereas education and self‐reported literacy were not.

**Conclusion:**

Participants with inflammatory arthritis generally held positive views about vaccination, although there was considerable uncertainty as to which vaccinations were recommended for them. This study highlights the need for improved consumer information about vaccination recommendations for people with inflammatory arthritis.

**SIGNIFICANCE & INNOVATIONS**

- This study of vaccination information sources and perception in people with inflammatory arthritis was uniquely conducted just prior to the COVID‐19 pandemic.
- Participants generally expressed positive vaccination views regarding safety, efficacy, and access. Vaccine hesitancy was primarily attributable to uncertainty and a perceived lack of information about which vaccines were recommended.
- Younger age, male gender, and having more concerns about the harms and overuse of medicines in general were associated with lower adherence and greater uncertainty about vaccinations. Of note, education and self‐reported literacy were not associated with vaccine hesitancy.
- Improving vaccination rates and reducing vaccine hesitancy requires improved consumer information for people with inflammatory arthritis.

## INTRODUCTION

People with autoimmune inflammatory arthritis, including rheumatoid arthritis (RA), psoriatic arthritis (PSA), and ankylosing spondylitis, are at increased risk of morbidity and mortality from vaccine‐preventable infections (1, 2, 3). This predisposition to more serious infection is multifactorial in the setting of disease‐related immune dysregulation, immunosuppressing medications, comorbidities, and potentially reduced vaccine responses (1). Vaccination is therefore an essential consideration in the health care of patients with inflammatory arthritis.

Despite the availability of recommendations addressing vaccination in patients with inflammatory arthritis, real‐world prescribing of vaccinations for this group requires balancing of multiple considerations to tailor recommendations to each individual patient. These include current disease modifying antirheumatic drugs (DMARDs), current disease activity, and comorbidities.

The aim of this study was to describe rates of vaccination and predictors of vaccination adherence in people with inflammatory arthritis. The secondary aim of the study was to determine perceptions and information sources related to vaccination in this group of patients.

## PATIENTS AND METHODS

The Australian Rheumatology Association Database (ARAD) is a voluntary Australian biologic registry established in 2001 to collect patient‐reported long‐term safety and other outcome data from patients with inflammatory arthritis (4, 5). Participants are referred by their treating rheumatologist from public hospital and private practice settings across Australia. Following written informed consent, participants complete a baseline ARAD questionnaire, with follow‐up questionnaires repeated every 6 months for 2 years, then at 12‐month intervals. At the time of this study, 69% of ARAD participants were opting to complete their usual questionnaires online. Participants with RA, PSA, or ankylosing spondylitis registered with ARAD who had completed an online ARAD questionnaire in the previous 12 months were invited to participate in an online survey. A link to the survey was sent by email to 1498 participants in January 2020, with a reminder sent 2 weeks later to those who had not yet responded. Of note, this study was conducted prior to the COVID‐19 pandemic. The survey link was closed 4 weeks after the initial email was sent. Study data were collected and managed using REDCap electronic data capture tools (6, 7) hosted at Monash University.

Data used were from annual ARAD questionnaires and included demographic information, the most recent updated medication use (prednisolone, DMARDs, biologic DMARDs [bDMARDs], opioid), smoking history (current/past), and comorbidities (hypertension, ischemic heart disease, diabetes, lung disease).

The survey (Supplementary File 1) collected information about most recent vaccinations of influenza, pneumococcal, zoster, and pertussis, and participants were asked whether they believed that they should have these vaccinations (Yes/No/Unsure).

Vaccination perceptions were assessed using a modified World Health Organization (WHO) Vaccination Hesitancy Scale (VHS) (8). The VHS is composed of 13 questions about perceived vaccination accessibility, safety, and efficacy and knowledge about vaccination recommendations. Participants responded “yes,” “no,” or “unsure” to each question. The original VHS questionnaire was developed to measure a parent's hesitancy about vaccination for their children; therefore, the wording of the questions was modified to reflect the participant's attitude toward vaccination for themselves.

In addition, participants were asked which sources they used for information about vaccinations. A list of possible information sources was provided for participants able to select more than one source. The sources listed were rheumatologists, rheumatology nurses, general practitioners (GPs), pharmacists, relatives, friends, other patients, educational internet sites (Eduweb), other internet sites (other web), internet chat‐rooms, social media, and mainstream media. If participants reported using a source, they were then asked to rate the advice provided by each resource regarding vaccination, ranging from very negative to very positive.

Respondents also completed the general Beliefs about Medicine Questionnaire (BMQ‐General) (9), which assesses beliefs about harms and overuse of medicines in general. Although the BMQ was originally developed and validated using responses from patients with nonrheumatic chronic disease, its use has been broadened to other settings, including patients with RA (10, 11, 12). Respondents indicated their level of agreement with each item in the questionnaire on a five‐point Likert scale (1 = strongly disagree to 5 = strongly agree). BMQ‐General harms and overuse scores were calculated by averaging scores within each of the harms (five questions) and overuse (three questions) domains for each participant. This grouping of subscale questions was previously validated in ARAD participant data (13), yielding Cronbach's alpha of 0.76 and 0.87, respectively, for the harms subscale and overuse subscale. The BMQ‐General scores therefore ranged between 1 and 5, with higher scores indicating the degree to which participants perceived medications to be harmful or overused, respectively.

The Single‐Item Literacy Screener (SILS) (14) was used to screen for limited reading ability, which is one component of health literacy. Participants were asked a single question as to how often they needed assistance reading health information materials, with answer options ranging from 1 (always) to 5 (never). Scores of 1 (always) or 2 (often) were considered to indicate some difficulty with reading printed health‐related material. When compared to the Short Test of Functional Health Literacy in Adults, the SILS had a sensitivity of 54% and a specificity of 83% (14).

### Statistical analysis

De‐identified data were analyzed in both Stata version 16 (StataCorp LLC, TX) and R version 3.6.3 (15).

Influenza vaccination adherence was defined as having received an influenza vaccination within the last 12 months, as yearly vaccinations are recommended for all Australians adults (Supplementary File 2).

Answers to the modified WHO VHS questions (no/unsure/yes) were analyzed using a distance‐based clustering approach to identify groups of participants. The clustering method involved partitioning of participants using a Gower distance matrix which is suitable for categorical data with a medoid iterative clustering procedure. Silhouette width, which is an aggregated measure of how similar an observation is to its own cluster compared with its closest neighboring cluster, was used to define the optimal number of clusters. Analysis was performed using R library “cluster” (16).

Tabulations and multivariable regression analyses for influenza adherence (logistic), VHS cluster membership (logistic), and the number of vaccination sources used (ordinal logistic) were performed in Stata. Predictors (covariates) for the regression analyses included age (scaled in decades), female gender, BMQ‐General harms and overuse scores, further education (defined as university, Technical and Further Education, or College of Advanced Education following high school), literacy help (defined as always/often requiring help with reading health‐related information from SILS), and current bDMARD/targeted synthetic DMARD (tsDMARD) use.

### Permissions

ARAD has ethics approval from Monash University and other sites, including Central Adelaide Local Health Network (CALHN). Permission for this study was obtained through the ARAD Steering Committee. Ethics approval for this study was granted by the CALHN Human Research Ethics Committee. Each participant gives consent to be enrolled in ARAD.

## RESULTS

### Study participants

The survey response rate was 66% (994/1498). The median age of respondents (62 years old) was 3 years older than nonresponders (59 years old) (*P* < 0.001). There were no significant differences with regard to gender, or disease between responders and nonresponders.

The respondents’ demographics, comorbidities, smoking status, and current medications are listed in Table 1. The majority of participants were female (666/994, 67%) with a median age of 62 years, and most (794/994, 80%) were currently on b/tsDMARD therapy. Many had completed further education following high school (644/994, 65%), and only 20 (2%) indicated some difficulty with reading printed health‐related materials.

**Table 1:** Demographic and clinical characteristics of survey respondents (N = 994)

Characteristic	Value
Females, N (%)	666 (67%)
Age (y), median (IQR)	62 (54‐69)
Age at diagnosis (y), median (IQR)	40 (30‐52)
Years on ARAD, median (IQR)	10 (5‐13)
Disease, N (%)	
Rheumatoid arthritis	648 (65%)
Spondyloarthropathy	180 (18%)
Psoriatic arthritis	166 (17%)
Education, N (%)	
Did not complete high school	127 (12%)
Completed high school	223 (22%)
University, TAFE, or CAE	644 (65%)
Smoking status, N (%)	
Current	41 (4%)
Ever (regular)	428 (44%)
BMQ‐General^a^, median (IQR)	
Harms	2.2 (1.8‐2.6)
Overuse	2.7 (2.0‐3.0)
Literacy screen^b^: help required, N (%)	
Always	10 (1%)
Often	10 (1%)
Sometimes	49 (5%)
Rarely	130 (13%)
Never	795 (80%)
Comorbidities, N (%)	
Hypertension	500 (50%)
Ischaemic heart disease	132 (13%)
Diabetes	110 (11%)
Lung disease	237 (24%)
Current medications	
Opioids	250 (25%)
bDMARDs	698 (70%)
tsDMARDs	96 (10%)
Methotrexate	495 50%
Leflunomide	85 (9%)
Prednisolone	238 (24%)

Abbreviations: ARAD, Australian Rheumatology Association Database; bDMARD, biologic disease modifying antirheumatic drug; BMQ‐General, Beliefs about Medicine Questionnaire (General); CAE, College of Advanced Education; DMARD, disease modifying antirheumatic drug; IQR, interquartile range; TAFE, Technical and Further Education; tsDMARD, targeted synthetic disease modifying antirheumatic drug.

^a^ Scored on a 1‐5 scale.

^b^ Single‐Item Health Literacy Screener.

### Self‐reported vaccination rates

**Figure 1:**
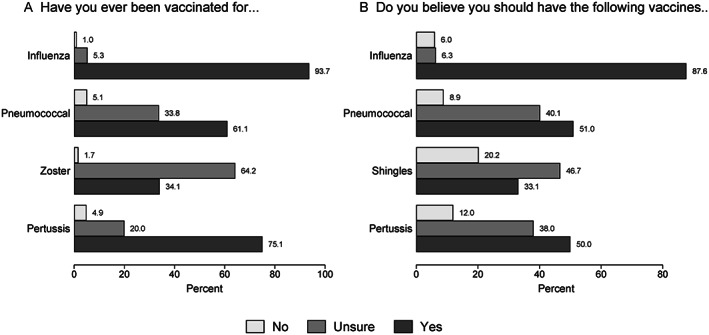
Participant responses (N = 994) to being asked whether, for specific vaccines, they had received the vaccine (in the last 5 years) (**A**) and whether they believed that they should have that vaccine (**B**).

Current Australian guidelines for vaccination schedules in adults with inflammatory arthritis are summarized in Supplementary File 2. Overall, most participants reported receipt of influenza vaccination in the last 5 years (931, 94%), followed by pertussis (746, 75%), pneumococcal (607, 61%), and zoster (339, 34%) vaccinations (Figure 1A), although a large number of participants (64%) were uncertain as to whether they had received the zoster vaccine. Most participants (871, 88%) believed that the influenza vaccine was necessary, but approximately 40% to 50% were unsure about the necessity of the others (Figure 1B). Because this survey was conducted in January 2020, there was no question about COVID‐19 vaccination.

Overall, influenza vaccination adherence, defined as a vaccine within the last 12 months, was high (828/994, 83%) but was highest in participants aged 65 years and older (360/405, 89%), compared to those aged less than 65 years (468/589, 79%), *P* < 0.001.

### Modified WHO VHS

Responses to the 13 questions for the modified WHO VHS (for any vaccine) are tabulated in Table 2. Participants generally held positive views regarding vaccination, with 90% reporting easy access to vaccinations (question K, Table 2), 82% reporting considering vaccines to be safe (question I, Table 2), and 93% reporting considering that vaccination could prevent serious infections (question H). However, only 43% were clear on which vaccinations were recommended for them (question M, Table 2).

**Table 2:** Responses to the modified WHO Vaccine Hesitancy Scale (for any vaccine), N = 994

Question	Yes	No	Unsure
Do you think vaccines are more important for people on certain medications?	795 (80%)	51 (5%)	148 (25%)
B If you have to spend more than one hour in travel time to get a vaccine do you consider it important enough to travel for?	829 (83%)	68 (7%)	97 (10%)
C Do you trust vaccine producers to provide safe and effective vaccines?	831 (84%)	37 (4%)	126 (13%)
D Have you ever decided not to get a vaccine for yourself?	219 (22%)	751 (76%)	24 (2%)
E Do you believe there are better ways to prevent diseases which are currently being prevented by vaccines?	65 (7%)	677 (68%)	252 (25%)
F Do you feel that you know which vaccines you should get for yourself?	491 (49%)	284 (29%)	219 (22%)
G Are you satisfied with your health‐professionals answers to your questions regarding immunisation?	859 (86%)	34 (3%)	101 (10%)
H Do you believe vaccines can prevent serious infections?	925 (93%)	13 (1%)	56 (6%)
I Do you believe vaccines are safe for you?	811 (82%)	34 (3%)	149 (15%)
J Do you feel you get enough information about vaccines and their safety?	698 (70%)	162 (16%)	134 (13%)
K Is access to vaccinations easy?	891 (90%)	28 (3%)	75 (8%)
L Do you feel confident that the general practice or hospital will have the vaccine you need, when you need them?	755 (76%)	63 (6%)	176 (18%)
M Do you know which vaccines are and aren't recommended for you?	421 (42%)	341 (34%)	232 (23%)

Of the 219 (22%) participants who had decided, on occasion, not to get a vaccination (question D, Table 2), 100 left a free text comment as to why they had made this decision. The most frequent response was being unable to have live vaccines due to immunosuppression (40%), which included 13% specifically commenting on the shingles (zoster) vaccine. A further 10% reported confusion over which vaccines were safe in the setting of their immunosuppression, 5% reported difficulty accessing vaccinations, and 4% had concerns regarding vaccine safety.

**Figure 2:**
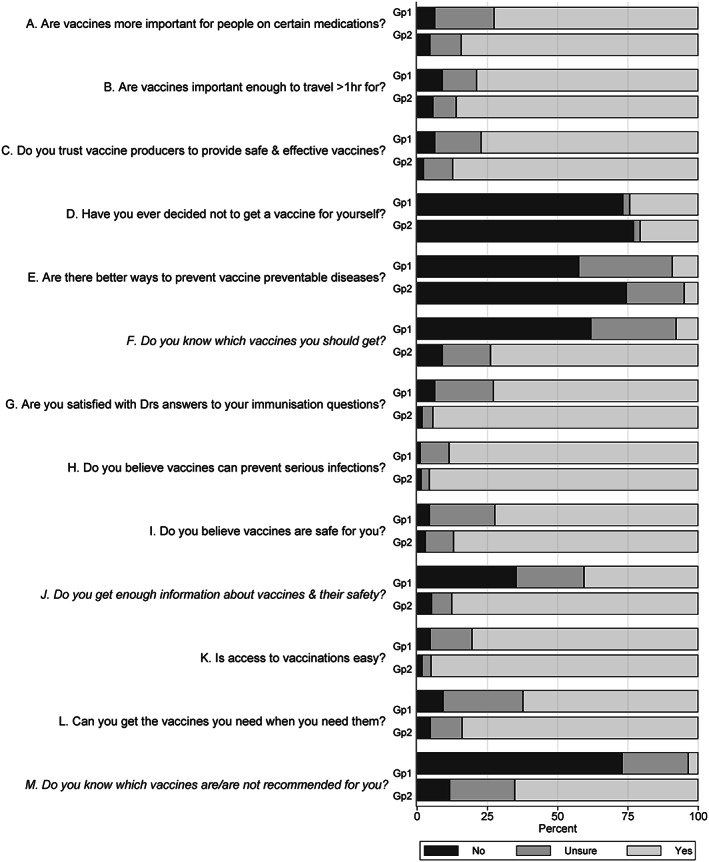
Participant subgroups derived from cluster analysis of responses to the modified WHO Vaccination Hesitancy Scale (Table 2). Two clusters were identified. Group 1 (Gp1) (n = 369, 37%) were more uncertain about vaccinations than Group 2 (Gp2) (n = 625, 63%), most notably in their responses to questions F, J, and M. WHO, World Health Organization.

Cluster analysis of the VHS responses identified two main groups of participants, Group 1 (369, 37%) and Group 2 (625, 63%). Comparative responses to each VHS question are illustrated in Figure 2 for each group, with Group 1 identified as the most vaccine hesitant. This vaccine hesitancy could primarily be attributed to a lack of understanding about which vaccines they should receive. Most participants in Group 1 (340/369, 92%) did not know, or were unsure, in response to question F (“Do you feel that you know which vaccines you should get for yourself?”), and 356/369 (96%) did not know, or were unsure, in response to question M (“Do you know which vaccines are and aren't recommended for you?”). Furthermore, only 150/369 (41%) of Group 1 participants responded positively to question J (“Do you feel you get enough information about vaccines and their safety?”) compared to 548/625 (88%) in Group 2. Compared to Group 2, influenza vaccination adherence was less likely in the more vaccine‐hesitant Group 1 (282/369 [76%] vs. 546/625 [87%], *P* < 0.001), although the influenza vaccination adherence in both cluster groups was high.

### Vaccination information sources

Participants reported consulting a number of different sources for vaccine information, in a variety of combinations, with the most frequent combination consisting of GPs and rheumatologists (179, 18%), followed by consultation of all 12 information sources (171, 17%). Overall, participants reported consulting a median of 3 (interquartile range 2‐7) vaccine information sources.

**Figure 3:**
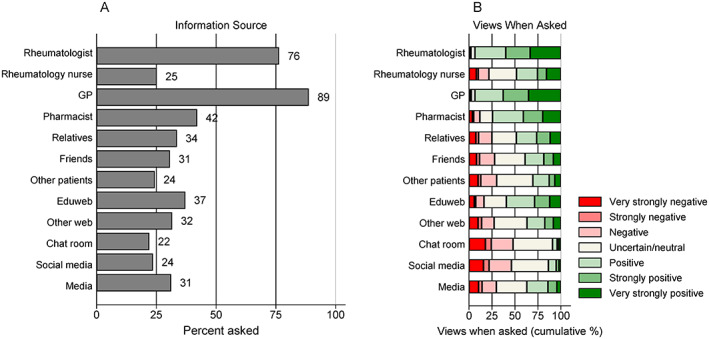
Vaccine information sources. The percentage of participants (n = 994) who reported consulting a specific information source about vaccination (**A**) and, when asked, whether the vaccination information was perceived to be positive or negative (**B**). “Eduweb” was specified as Internet Educational websites (eg Australian Rheumatology Association, Arthritis Australia); “Other web” as other internet websites; “Chat room” as internet chat rooms or forums; “Social media” as Twitter, Facebook, etc; and “Media” as newspapers, magazines, television. GP, general practitioner.

With the exception of rheumatology nurses, who are few in number and thus not widely accessible in Australia, health professionals were the most widely used information sources (Figure 3A). GPs were the most widely consulted information source (882, 89%), followed by rheumatologists (759, 76%) and pharmacists (418, 42%). These health professionals were also the most likely to provide positive advice about vaccinations (Figure 3B) with 825/882 (94%) participants reporting positive advice from GPs, 710/759 (94%) reporting positive advice from rheumatologists, followed by 311/418 (74%) reporting positive advice from pharmacists. Of those who received vaccination advice from rheumatology nurses, only 48% of participants reported the advice being positive.

Of the remaining information sources, educational websites were the most frequently used (369, 37%), with 219/369 (59%) of participants reporting positive vaccination advice. Participants were less likely to report positive vaccination advice from other information sources, particularly social media (20/234, 9%) and chat rooms (20/219, 9%). However, in general, participants were also less sure about their interpretation of the advice obtained from these other sources (Figure 3B).

### Covariate predictors of vaccination outcomes

**Figure 4:**
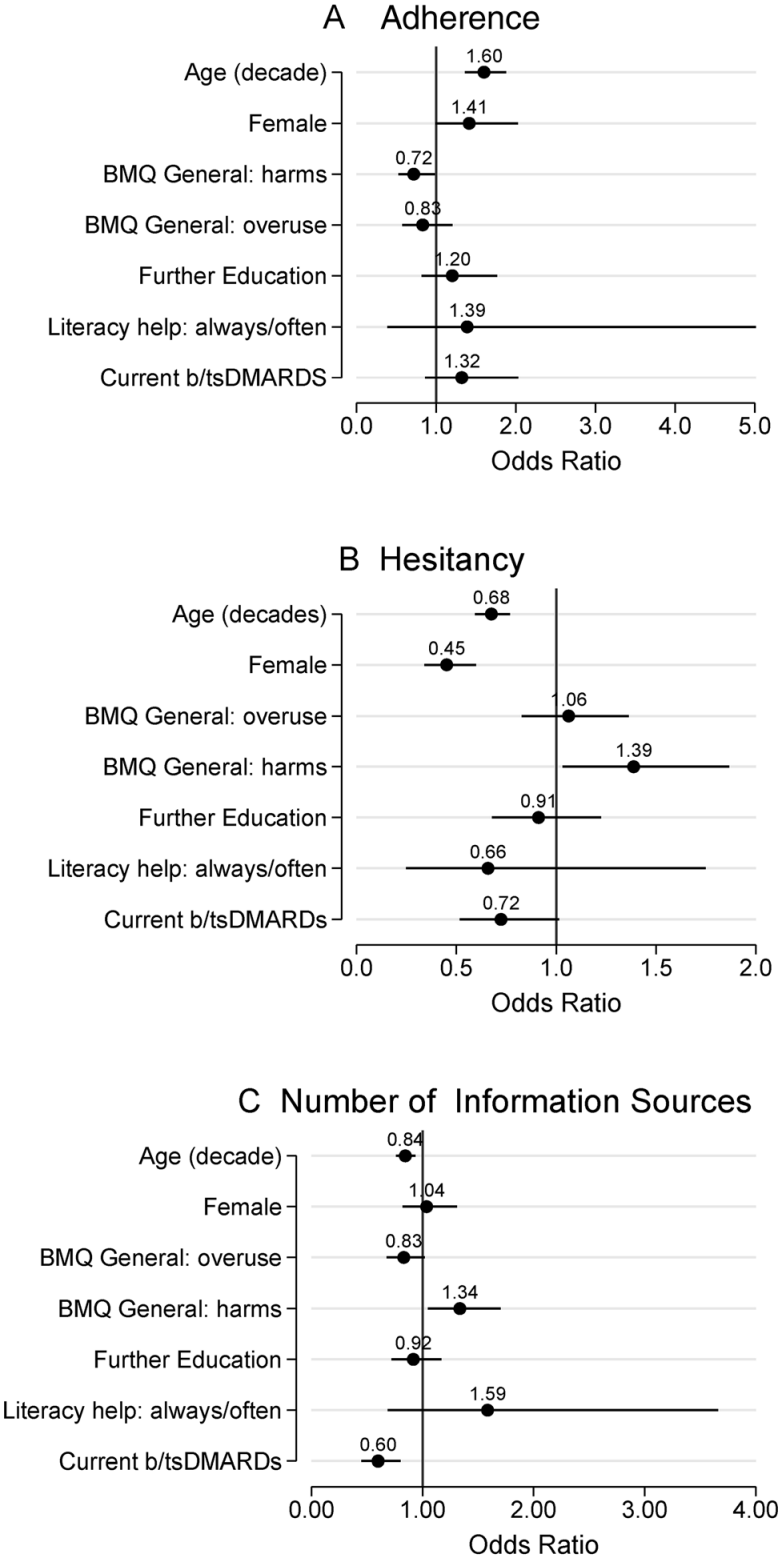
Coefficient plots for predictor variables for influenza vaccination adherence (yes vs. no) (**A**), vaccine hesitancy (Group 1 vs. Group 2 participants determined from the cluster analysis of the WHO Vaccination Hesitancy Scale) (**B**), and the number of vaccination information sources used (**C**). Panels (A) and (B) were analyzed by logistic regression and Panel (C) by ordinal logistic regression. Results are expressed as odds ratios with vertical bars indicating 95% confidence intervals. Further education was defined as education/training following high school. Literacy help was defined from responses to the Single‐Item Literacy Screener. bDMARD, biologic disease modifying antirheumatic drug; BMQ, Beliefs about Medicine (General); tsDMARD, targeted synthetic disease modifying antirheumatic drug; WHO, World Health Organization.

Multivariable regression analysis results are shown as coefficient plots for influenza vaccination adherence (Figure 4A), vaccine hesitancy (Group 1 vs. Group 2 identified from the VHS cluster analysis, Figure 4B), and the number of utilized vaccination information sources (Figure 4C).

Older age (scaled in decades) was associated with increased influenza vaccination (odds ratio [OR] 1.6, 95% CI: 1.4‐1.9), less vaccine hesitancy (OR 0.67, 95% CI: 0.59‐0.77), and use of fewer vaccination information sources (OR 0.84, 95% CI: 0.76‐0.94). Females were more likely to receive vaccination (OR 1.4, 95% CI: 0.99‐2.0) and less likely to be vaccine hesitant (OR 0.45, 95% CI: 0.34‐0.60), whereas current b/tsDMARD users were less likely to be vaccine hesitant (OR 0.72, 95% CI: 0.52‐1.0) and used fewer vaccination information sources (OR 0.60, 95% CI: 0.45‐0.80).

Participants’ general beliefs about medicine also influenced their vaccination outcomes. Higher BMQ‐General overuse scores were associated with lower vaccination (OR 0.72, 95% CI: 0.52‐0.98) and, perhaps paradoxically, fewer used information sources (OR 0.83, 95% CI: 0.68‐1.0). Higher BMQ‐General harms scores were associated with more vaccine hesitancy (OR 1.4, 95% CI: 1.0‐1.9) and more information sources used (OR 1.3, 95% CI: 1.0‐1.7).

Of note, neither further education nor requiring help reading health‐related materials was associated with any of the vaccination outcomes.

## DISCUSSION

This study conducted in January 2020 provides unique data with measurement of vaccination perceptions and information sources prior to the current COVID‐19 pandemic. Overall, participants with inflammatory arthritis generally held positive views about vaccination, although there was considerable uncertainty as to which vaccinations were recommended for them.

Vaccinations are generally recommended in people with inflammatory arthritis due to increased risk of morbidity and mortality from vaccine‐preventable infections. Yet vaccine efficacy may be lower because of immunosuppressive treatment, which may be offset by the timing of vaccination in relation to treatment. Although scientific evidence for the safety and effectiveness of large‐scale vaccination campaigns is well established (17), vaccine hesitancy is of increasing concern.

We examined three aspects of vaccinations in people with inflammatory arthritis: vaccination rates, attitudes to vaccination (VHS), and sources of vaccine information used by patients. Key messages are that although vaccination rates were high, many participants expressed uncertainty about which vaccinations they should receive. Uncertainty was also manifest in the number of utilized vaccine information sources. Risk factors for lower adherence or more uncertainty included younger age, male gender, and general beliefs about medicine (particularly concern about harms). Of interest, education and self‐reported literacy issues were not key factors. Reassuringly, rheumatologists and GPs (the primary source of vaccine delivery) were the most frequently used source of positive information regarding vaccinations for patients, with participants often unsure how to interpret information from other sources. Although fewer patients in Australia have access to specialized rheumatology nurses, it was of concern that, of those who did consult a rheumatology nurse regarding vaccination, there was much lower positive perception of vaccine information compared to GPs and rheumatologists.

This was a large national study, with an excellent response rate, that provides comprehensive information about vaccinations in patients with inflammatory arthritis. Participants are cared for in a variety of settings, including both public hospital clinics and private clinics. However, there are several limitations. Firstly, the participants are unlikely to be completely representative of the overall inflammatory arthritis population. ARAD was initially established as a registry to assess safety and efficacy of biologic agents in inflammatory arthritis. The high proportion of participants with current b/tsDMARD and opioid use reflect that, as expected, this is a cohort with moderate to severe disease. Furthermore, the participants are quite well educated, with good health literacy skills, which may be expected in a cohort who complete an annual online questionnaire. Collectively, this ARAD cohort may be well informed about the benefits and risks of vaccinations and therefore skewed toward a more favorable view. Secondly, receipt of vaccines was self‐reported, which relied on accurate patient recall. Furthermore, this survey was conducted prior to the COVID‐19 pandemic; therefore, vaccine attitudes generally may have been impacted by the amount of both positive and negative commentary related to the COVID‐19 vaccine. Finally, the questions regarding timing of vaccination rates did not provide enough scope to assess whether a patient was fully up to date with vaccination and whether they had received the correct recommended vaccines over the appropriate time course. For example, although participants were able to report they had received the pneumococcal vaccination, they were not asked whether they had completed the recommended course (13‐valent pneumococcal conjugate vaccine followed by several doses of the 23‐valent pneumococcal polysaccharide vaccine). With the data available, we were also not able to assess whether participants who had previously received pertussis vaccination were up to date with a booster vaccine within the past 10 years or whether patients had been offered and received the Zoster vaccine prior to commencement of the b/tsDMARD therapy.

Identifying the factors that contribute to vaccine hesitancy and barriers to vaccination adherence is of particular current relevance, as the uptake of vaccination for COVID‐19 during the current global pandemic has been hindered by vaccine hesitancy and misinformation campaigns. COVID‐19 vaccines are currently recommended for Australians with inflammatory arthritis (18, 19), although there is, as yet, limited evidence on the safety and efficacy of these vaccinations for patients with inflammatory arthritis. Strikingly, a recent multinational survey of 1258 patients with autoimmune and inflammatory diseases identified 86% as hesitant or suspicious about receiving the COVID‐19 vaccine (20). Our study was conducted prior to the onset of the COVID‐19 pandemic, and our on‐going research will examine differences and similarities in attitudes and adherence between COVID‐19 vaccines and other recommended vaccines, and whether misinformation about COVID‐19 has subsequently influenced views about other vaccinations.

Studies are required to determine effective interventions for improving vaccination adherence. A recent US study, evaluating multimodal interventions addressing patient communication with their health care provider and vaccine access, demonstrated an improvement in influenza vaccine adherence from 49% to 63% in patients with RA (21). It is worth noting that, in this Australian study of participants with inflammatory arthritis, in whom the influenza vaccination adherence rate was high (83%), nearly all participants reported discussing vaccinations with their rheumatologist, and many vaccines are provided free to eligible people through the National Immunisation Program.

This study has demonstrated that, although Australian patients with inflammatory arthritis view vaccinations in a mostly positive light, many are uncertain about which vaccinations are recommended for them or feel they have insufficient information. This highlights the need for improved consumer information about vaccinations for people with inflammatory arthritis, and rheumatologists have a key role in the provision of education and vaccination advice to both patients and their GPs.

## AUTHOR CONTRIBUTIONS

All authors were involved in drafting the article or revisiting it critically for important intellectual content, and all authors approved the final version to be published. Dr. Catherine Hill had full access to all of the data in the study and takes responsibility for the integrity of data and the accuracy of the data analysis.

### Study conception and design

Lyon, Quinlivan, Lester, Barrett, Whittle, Rowett, Black, Sinnathurai, March, Buchbinder, Hill.

### Acquisition of data

Quinlivan, Lester, Barrett, Whittle, Black, March, Buchbinder.

### Analysis and interpretation of data

Lyon, Lester, Rowett, Black, Buchbinder.

## Supporting information


**File 1:** Supplementary File 1

**File 2:** Supplementary File 2
