# Supplementary material for: Vaccination Rates, Perceptions, and Information Sources Used by People With Inflammatory Arthritis
Source: ACR Open Rheumatol. 2023 Jan 18;5(2):84–92. doi: 10.1002/acr2.11525 (PMC9926066; doi:10.1002/acr2.11525)
Supplement: Supplementary file 1 — File 1: Supplementary File 1 [file ACR2-5-84-s001.pdf]

5. YOUR VIEWS ABOUT MEDICINES IN GENERAL

5.1 These are statements that other people have made about medicines in general.
Please show how much you agree or disagree with them by ticking the appropriate box

	Strongly Agree	Agree	Uncertain	Disagree	Strongly Disagree
a) Doctors use too many medicines	☐	☐	☐	☐	☐
b) People who take medicines should stop their treatment for a while every now and again	☐	☐	☐	☐	☐
c) Most medicines are addictive	☐	☐	☐	☐	☐
d) Natural remedies are safer than medicines	☐	☐	☐	☐	☐
e) Medicines do more harm than good	☐	☐	☐	☐	☐
f) All medicines are poisons	☐	☐	☐	☐	☐
g) Doctors place too much trust on medicines	☐	☐	☐	☐	☐
h) If doctors had more time with patients they would prescribe fewer medicines	☐	☐	☐	☐	☐

5.2 Comments

6. YOUR VIEWS ABOUT VACCINATION

We would like to ask you about your personal views about VACCINATION
These are statements other people have made about their medicines
Please show how much you agree or or disagree with them by ticking the appropriate box
There are no right or wrong answers. We are interested in your personal views

6.1 When was the last time you had this vaccine?

	Within the last 12 months	12-24 months ago	More than 5 years ago	3-5 years ago	Unsure	I have never had this vaaccine
a) Influenza vaccination (or Fluvax)	☐	☐	☐	☐	☐	☐
b) Pneumococcal vaccination	☐	☐	☐	☐	☐	☐
c) Shingles vaccination	☐	☐	☐	☐	☐	☐
d) Whooping cough (pertussis)	☐	☐	☐	☐	☐	☐

6.2 Where did you have the influenza vaccination?

☐ GP ☐ Specialist ☐ Hospital ☐ Pharmacy ☐ Work ☐ Other ☐ Not sure

6. YOUR VIEWS ABOUT VACCINATION

6.3 Do you believe you should have this vaccine?

	Yes	No	Unsure
Influenza vaccination (or Fluvax)	☐	☐	☐
Pneumococcal vaccination	☐	☐	☐
Shingles vaccination	☐	☐	☐
Whooping cough (pertussis)	☐	☐	☐

6.4 Have you ever had an episode of shingles? ☐ Yes ☐ No ☐ Not sure

If YES → **6.4.1** Was it before or after receiving the shingles vaccine? ☐ Before ☐ After ☐ Not sure

6.5 Comments

6.6 Views about Vaccination

	No	Yes	Not sure
a) Do you think vaccines are more important for people on certain medications?	☐	☐	☐
b) If you have to spend more than one hour in travel time to get a vaccine do you consider it mportant enough to travel for?	☐	☐	☐
c) Do you trust vaccine producers to provide safe and effective vaccines?	☐	☐	☐
d) Have you ever decided not to get a vaccine for yourself?	☐	☐	☐
e) Do you believe there are better ways to prevent diseases which are currently being prevented by vaccines?	☐	☐	☐
f) Do you feel that you know which vaccines you should get for yourself?	☐	☐	☐
g) Are you satisfied with your health-professionals answers to your questions regarding immunisation?	☐	☐	☐
h) Do you believe vaccines can prevent serious infections?	☐	☐	☐
i) Do you believe vaccines are safe for you?	☐	☐	☐
j) Do you feel you get enough information about vaccines and their safety?	☐	☐	☐
k) Is access to vaccinations easy?	☐	☐	☐
l) Do you feel confident that the general practice or hospital will have the vaccine you need when you need them?	☐	☐	☐
m) Do you know which vaccines are and aren't recommended for you?	☐	☐	☐

6.7 Comments

6. YOUR VIEWS ABOUT VACCINATION

6.8 Do you remember any events in the past that would have discouraged you from getting a vaccine?

6.9 Where did you get your advice and information from AND was this advice positive?

	Not asked	Very Strongly Positive	Strongly Positive	Positive	Uncertain / Neutral	Negative	Strongly Negative	Very Strongly Negative
a Rheumatologist	☐	☐	☐	☐	☐	☐	☐	☐
b) Rheumatology nurse	☐	☐	☐	☐	☐	☐	☐	☐
c) General Practitioner	☐	☐	☐	☐	☐	☐	☐	☐
d) Pharmacist	☐	☐	☐	☐	☐	☐	☐	☐
e) Relative	☐	☐	☐	☐	☐	☐	☐	☐
f) Friends	☐	☐	☐	☐	☐	☐	☐	☐
g) Other patients	☐	☐	☐	☐	☐	☐	☐	☐
h) Internet Educational websites (eg Australian Rheumatology Association / Arthritis Australia)	☐	☐	☐	☐	☐	☐	☐	☐
i) Other internet websites	☐	☐	☐	☐	☐	☐	☐	☐
j) Internet chat rooms/forums	☐	☐	☐	☐	☐	☐	☐	☐
k) Social media (Twitter, facebook etc)	☐	☐	☐	☐	☐	☐	☐	☐
l) Media (newspapers, magazines, television)	☐	☐	☐	☐	☐	☐	☐	☐

6.10 Comments

7. OTHER MEDICATIONS

We are intered in the other arthritis medications that you are on

7.1 Methotrexate

☐ I am currently taking methotrexate → **7.1.1** Dose ____ **7.1.2** Adminstered ☐ Oral/Tablet ☐ Injection

☐ I used to take methotrexate but it was stopped → **7.1.3** Reason
- ☐ Didn't work
- ☐ Side effects
- ☐ Didn't want to take it anymore
- ☐ Other → **7.1.3.1** Specify ____

☐ It was offered to me but I decided not to take it → **7.1.4** Reason ____

☐ None of the above

7.2 Leflunomide

☐ I am currently taking leflunomide → **7.2.1** Dose ☐ 10mg ☐ 20mg ☐ Other **7.2.1.1** Specify ____

☐ I used to take leflunomide but it was stopped → **7.2.2** Reason
- ☐ Didn't work
- ☐ Side effects
- ☐ Didn't want to take it anymore
- ☐ Other → **7.2.2.1** Specify ____

☐ It was offered to me but I decided not to take it → **7.2.3** Reason ____

☐ None of the above

7.3 Prednisolone

☐ I am currently taking prednisolone → **7.3.1** Daily Dose ____

7.3.2 How many days per week do you take it ____

7.3.3 What year were you started on prednisolone ____

☐ I used to take prednisolone but it was stopped → **7.3.4** Reason
- ☐ Didn't work
- ☐ Side effects
- ☐ Didn't want to take it anymore
- ☐ Other → **7.3.4.1** Specify ____

☐ It was offered to me but I decided not to take it → **7.3.5** Reason ____

☐ None of the above

7.4 How ofter do you need to have someone help you when you read instructions, pamphlets, or other written material from your doctor or pharmacy?

☐ Always ☐ Often ☐ Sometimes ☐ Rarely ☐ Never
