# Supplementary material for: Vaccination Rates, Perceptions, and Information Sources Used by People With Inflammatory Arthritis
Source: ACR Open Rheumatol. 2023 Jan 18;5(2):84–92. doi: 10.1002/acr2.11525 (PMC9926066; doi:10.1002/acr2.11525)
Supplement: Supplementary file 2 — File 2: Supplementary File 2 [file ACR2-5-84-s002.pdf]

Supplementary File 2: Recommendation for Vaccinations of patients (>18 years) with autoimmune inflammatory rheumatic diseases (AIIRD) in Australia

**Vaccine**	**Recommendation**
Influenza	Recommended annually for all patients with AIIRD
Pneumococcal (Pneumovax-23, Prevenar-13)	Recommended for immunosuppressed patients
SARS-Cov-2 (Comirnaty Vaxzevria, Nuvaxovid Spikevax)	Two course primary vaccination course and single booster recommended for all people >16 years<br>Two course primary vaccination course and two boosters for mild-moderately immunocompromised people and those aged >50 years.<br>Three course primary vaccination course and two boosters in severely immunocompromised peopl
Hepatitis B (Engerix B)	Recommended for immunosuppressed patients
Human papilloma virus (Gardasil 9)	Recommended for immunosuppressed patients
Diphtheria, tetanus, pertussis (dTpa) (Boostrix)	Recommendations as per the general population<br>dTpa: Pregnant woman, adults with incomplete childhood vaccination, people 50 years old or >65 years without a tetanus vaccine in past 10 years,<br>Additional recommendations for pertussis vaccine: people working in early childhood education, caring for babies under 6 months old or healthcare workers.
Poliomyelitis (Ipol)	Recommendations as per the general population<br>Adults with incomplete childhood vaccination, healthcare and laboratory workers with potential for exposure to polivirus
Herpes zoster (Zostavax*, Shingrix)	Consider in people aged ≥50 years with AIIRD. Recommended in all adults ≥60 years.<br>NB: Shringrix is the recommended zoster vaccine in immunocompromised adults >18 years old. Zostavax* is an alternative in adults ≥60 years old in whom live vaccines are not contraindicated
Varicella (Varilix*, Varivax*)	Consider if no serological evidence of previous varicella zoster infection and live vaccines are not contraindicated
Measles, mumps and rubella (MMR II*, Priorix*)	Consider in patients with AIIRD on low dose immunosuppressive therapy in whom live vaccines are not contraindicated

*Live vaccines: Contraindicated in patients on bDMARDs, tsDMARDs or other significant immunosuppression (equivalent to prednisolone ≥20mg/day, methotrexate ≥0.4 mg/kg/week, azathioprine ≥3mg/kg/day)

Table adapted from Australian Rheumatology Association Recommendations for vaccination of patients (>18 years) with autoimmune inflammatory rheumatic diseases (AIRD) in Australia" and the Australian Immunisation Handbook

References:

1. Australian Technical Advisory Group on Immunisation (ATAGI), Australian Immunisation Handbook. 2018, Australian Government Department of Health, immunisationhandbook.health.gov.au, Canberra.
2. Australian Rheumatology Association. Recommendations for vaccination of patients (>18 years) with autoimmune inflammatory rheumatic diseases (AIRD) in Australia. July 2021. (URL: https://rheumatology.org.au/Portals/2/Documents/Public/Professionals/Vaccination%20Information/210802%20Table%20of%20Vaccinations%20new%20branding.pdf?ver=2022-02-16-090913-117)
